# A long-term culture model for investigating senescence-associated dysregulation in macrophages

**DOI:** 10.3389/fimmu.2025.1661497

**Published:** 2025-10-02

**Authors:** Andy Ruiz, María Guadalupe Lucero-Gil, Martha Torres, Esmeralda Juárez

**Affiliations:** ^1^ Laboratorio de Alta Contención Biológica (LACBio), Instituto Nacional de Enfermedades Respiratorias Ismael Cosío Villegas, Mexico City, Mexico; ^2^ Facultad de Ciencias, Universidad Nacional Autónoma de México, Mexico City, Mexico; ^3^ Laboratorio de Inmunobiología de la Tuberculosis (LIT), Instituto Nacional de Enfermedades Respiratorias Ismael Cosío Villegas, Mexico City, Mexico

**Keywords:** senescence, macrophages, immune dysregulation, inflammation, M1 polarization, spectral flow cytometry

## Abstract

Macrophage senescence is increasingly recognized as a key contributor to immune dysfunction during aging and chronic inflammation. Here, we developed a reproducible long-term *in vitro* culture model to investigate the senescence’s phenotypic and functional consequences in human macrophages. Monocyte-derived macrophages (MDMs) were cultured for 7, 14, or 21 days and assessed for canonical senescence markers and immune function. While macrophages at day 7 exhibited minimal expression of β-galactosidase, H2AXpS139, and CDKN2A (p16INK4a), these markers were significantly upregulated at days 14 and 21, indicating progressive acquisition of a senescent phenotype. Cell viability remained above 95% across all time points, confirming the system’s stability. High-dimensional flow cytometry and unsupervised clustering revealed marked phenotypic remodeling over time, shifting from anti-inflammatory CD163^+^/CD206^+^ profiles to proinflammatory CD14^+^/CD64^+^/TLR2^+^ populations. Five distinct macrophage subpopulations were identified, each with dynamic temporal distribution and unique marker expression, highlighting a loss of plasticity and emergence of senescence-associated states. Functionally, day 7 macrophages produced a diverse cytokine panel responding to lipopolysaccharide (LPS), including IL-2, IL-12p70, IL-4, IL-5, TNF-α, GM-CSF, IFN-γ, and IL-10. In contrast, macrophages at days 14 and 21 displayed a markedly restricted cytokine profile, retaining the secretion of cytokines but significantly downregulating the secretion of Th1-type cytokines. This long-term culture model constitutes a robust, physiologically relevant tool to study immune aging, senescence-driven immune reprogramming, and inflammation in macrophages with the potential for the preclinical evaluation of therapeutic strategies to ameliorate inflammaging and restore immune competence.

## 1 Introduction

The global rise in life expectancy has led to a parallel increase in the prevalence of age-associated diseases, including chronic inflammatory conditions, cancer, neurodegenerative disorders, and recurrent infections. A critical factor underpinning these diseases is the progressive deterioration of immune function with age, collectively referred to as immunosenescence (1). This process encompasses reduced responsiveness of both innate and adaptive immune compartments, leading to impaired pathogen clearance, altered tissue repair mechanisms, and a chronic proinflammatory state. Among the cellular contributors to immunosenescence, senescent cells play a central role. These are cells that undergo permanent cell cycle arrest in response to a range of stressors, including oxidative stress, telomere shortening, and DNA damage (2–5).

As innate immune sentinels, macrophages orchestrate responses to infection, injury, and tissue homeostasis. Their remarkable plasticity enables them to polarize toward either proinflammatory (M1) or anti-inflammatory (M2) phenotypes depending on environmental stimuli (6, 7). However, the induction of senescence in macrophages and its implications for immune regulation remain incompletely understood. Emerging evidence suggests that senescent macrophages exhibit impaired phagocytic capacity, altered polarization, and a proinflammatory senescence-associated secretory phenotype (SASP), all of which may exacerbate chronic inflammation and contribute to inflammaging (5, 8–13).

Senescent cells are defined by a characteristic set of biomarkers, including increased senescence-associated β-galactosidase (SA-β-gal) activity, upregulation of CDKN2A (p16INK4a) and CDKN1A (p21CIP1), activation of DNA damage response pathways such as histone H2AX phosphorylation (H2AXpS139), and secretion of a complex milieu of cytokines, chemokines, and proteases collectively referred to as SASP (10, 11, 13–16). The SASP can reinforce senescence through autocrine and paracrine signaling, reshape tissue microenvironments, and promote immune dysregulation by altering leukocyte recruitment and function.

While most senescence studies have focused on proliferative cells such as fibroblasts or lymphocytes, a scarcity of models addresses the induction and impact of senescence in macrophages. This gap hinders our understanding of innate immune dysfunction during the aging process. Furthermore, translational studies in elderly individuals are limited by ethical and logistical constraints related to sample collection, particularly the volume of peripheral blood required to obtain sufficient monocytes for macrophage differentiation (17, 18).

To address this, we established a robust *in vitro* model of macrophage senescence by extending standard monocyte-derived macrophage (MDM) cultures from 7 days (D7) to 14 (D14) and 21 days (D21). This extended culture protocol resulted in the progressive induction of canonical senescence markers and altered functional states, without the need for additional chemical or physical stressors. Using this model, we performed a comprehensive phenotypic and functional analysis, including quantification of SA-β-gal activity, expression of CDKN2A and H2AXpS139, surface marker profiling by high-dimensional flow cytometry (e.g., CD14, CD64, CD206, CD163, TLR2, HLA-DR), and assessment of cytokine responses following lipopolysaccharide (LPS) stimulation.

The primary aim of this study was to provide a physiologically relevant model for studying macrophage senescence and its implications for immune dysregulation during aging. By identifying phenotypic transitions, secretory dysfunctions, and potential regulatory targets, this model offers a valuable tool for investigating the role of innate immune senescence in age-related inflammatory diseases and guiding future therapeutic interventions aimed at mitigating immunosenescence and inflammaging.

## 2 Materials and methods

### 2.1 Monocyte isolation and macrophage differentiation

Peripheral blood mononuclear cells (PBMCs) were isolated from leukocyte-enriched buffy coats obtained from six healthy adult donors (ages 20–35) with no chronic inflammatory or infectious disease history. Samples were provided by the blood bank of the Instituto Nacional de Enfermedades Respiratorias Ismael Cosío Villegas. The institutional review boards approved the study and waived the requirement for informed consent. PBMCs were separated by density gradient centrifugation using Lymphoprep™ (Axis Shield, Germany). CD14^+^ monocytes were subsequently purified from PBMCs using CD14 MicroBeads (Miltenyi Biotec, US), achieving a purity >95% as confirmed by flow cytometry.

Purified monocytes were seeded in ultra-low attachment 24-well plates (Corning, US) at a density of 1 × 10^6^ cells/mL and cultured in RPMI 1640 medium (Lonza, US) supplemented with 2 mM L-glutamine (Lonza), 10% heat-inactivated fetal bovine serum (FBS, Lonza), and 1% penicillin-streptomycin (Lonza) and cultured for 7 days (D7) to allow monocyte-to-macrophage differentiation (MDM) under standard culture conditions (37 °C, 5% CO_2_). To induce senescence, cultures were extended to 14 (D14) and 21 days (D21), with medium refreshing every 72 hours to maintain nutrient availability and prevent excessive cell stress unrelated to replicative or metabolic aging.

### 2.2 Stimulation with lipopolysaccharide

To assess functional responses, MDM were cultured for 7, 14, and 21 days as described above, and stimulated with 200 ng/mL lipopolysaccharide (LPS, from Escherichia coli O111: B4; Sigma-Aldrich, US) for 24 hours under standard culture conditions. Unstimulated controls were maintained in parallel for each time point to enable direct comparison of activation-dependent phenotypic and functional changes.

### 2.3 Senescence marker detection

Senescence-associated β-galactosidase (SA-β-gal) activity was evaluated using the CellEvent™ Senescence Green Flow Cytometry Assay Kit (Thermo Fisher Scientific, US), which detects increased lysosomal β-galactosidase activity, a hallmark of cellular senescence. After harvesting, cells were fixed in 2% paraformaldehyde (Sigma-Aldrich) for 15 minutes at room temperature and then washed twice with phosphate-buffered saline (PBS, Lonza) containing 1% bovine serum albumin (BSA, Sigma-Aldrich). The cell pellet was resuspended in the kit’s staining solution diluted 1:100 in PBS and incubated for 2 hours at 37 °C, protected from light. Following incubation, cells were washed and immediately analyzed for the hydrolysis of β-galactosidase by flow cytometry. Fluorescence was excited using a 488 nm blue laser and detected through a 530/30 nm bandpass filter. Data was acquired on a BD FACS Discover S8 (BD Biosciences, US) spectral flow cytometer and analyzed using FlowJo software (BD Biosciences). All reagents, antibodies, kits, and software used in this study are fully listed in **Supplementary Tables 1, 2**, including brand, catalog number, and identifier when available.

### 2.4 Surface and intracellular phenotyping

Cell viability was evaluated using the LIVE/DEAD™ Fixable Dead Cell Stain Kit (Thermo Fisher Scientific, US) at a 1:1000 dilution in phosphate-buffered saline (PBS) following the manufacturer’s protocol. After viability staining, surface marker staining was performed at 4 °C for 30 minutes in the presence of BD Horizon™ Brilliant Stain Buffer (BD Biosciences) to minimize fluorochrome spillover and spectral overlap. Cells were incubated with fluorochrome-conjugated monoclonal antibodies to CD3, CD14, CD64, CD163, CD206, HLA-DR, TLR2 (CD282), and TLR9 (CD289), as detailed in **Supplementary Table 1**. After surface staining, cells were fixed and permeabilized using the BD Cytofix/Cytoperm™ solution (BD Biosciences) and washed with BD Perm/Wash™ buffer. Intracellular staining for CDKN2A/p16-INK4a, phosphorylated H2AX (H2AXpS139), and the nuclear dye 7-AAD was conducted for 60 minutes at 4 °C in the dark using specific antibodies.

### 2.5 Spectral flow cytometry acquisition

Samples were acquired using the FACS Discover S8 spectral flow cytometer (BD Biosciences) and analyzed with FACS Chorus software. Fluorophore selection was optimized based on spectral resolution, antigen density, and fluorescence intensity, incorporating parameters such as Similarity Index (SI), Cross Stain Index (CSI), and Complexity Index (CI) to ensure accurate detection in high-dimensional panels. Daily quality control and calibration were performed using BD Spectral Setup Beads to maintain instrument performance. Spectral unmixing was carried out using a reference matrix generated from single-stained compensation controls and unstained samples, allowing for practical autofluorescence subtraction and precise deconvolution of overlapping emission spectra.

### 2.6 Phenotypic and multiparametric analysis

Flow cytometry data were analyzed using FlowJo™ v10.10 software (BD Biosciences). Initial preprocessing included gating on singlets, live cells, and CD14^+^ macrophages to ensure data quality. Single-marker expression was visualized through histogram plots and fluorescence intensity overlays across time points. High-dimensional analysis was conducted using t-distributed stochastic neighbor embedding (t-SNE) for dimensionality reduction and FlowSOM for unsupervised clustering, enabling the identification of phenotypically distinct macrophage subpopulations (19). Cleaned FCS files were analyzed independently for each time point (D7, D14, D21). Cluster validation and manual annotation were performed using X-shift clustering algorithms when needed; for functional comparisons following LPS stimulation, T-REX (Tracking Responders Expanding) was employed to identify significantly expanded or contracted phenotypic regions (20), enhancing the interpretation of dynamic immune responses. This unsupervised machine learning tool identifies phenotypic regions that expand or contract in response to specific conditions by comparing paired samples, such as unstimulated (medium) and LPS-stimulated cells.

### 2.7 Cytokine profiling

Cell-free culture supernatants were harvested 6 hours after LPS stimulation and immediately stored at –80 °C until analysis. Concentrations of IL-2, IL-4, IL-5, IL-10, IL-12p70, IFN-γ, TNF-α, and GM-CSF were quantified using the Bio-Plex Pro™ Human Cytokine Panel (Bio-Rad, US), following the manufacturer’s protocol. Fluorescent bead-based multiplex assays were run on the Bio-Plex™ 200 system, and data were processed using Bio-Plex Manager™ software for cytokine quantification. Cytokine production below the detection limits established by the manufacturer (**Supplementary Table 3**) was considered as zero.

### 2.8 Statistical analysis

Statistical analyses were conducted using GraphPad Prism version 10 (GraphPad Software, US). The Mann–Whitney U test was applied for comparisons between independent groups, while the Wilcoxon signed-rank test was used for paired data. A p-value < 0.05 was considered statistically significant. Specific statistical tests and sample sizes are indicated in the corresponding figure legends.

## 3 Results

### 3.1 Long-term culture induces senescence in human macrophages

**Figure 1:**
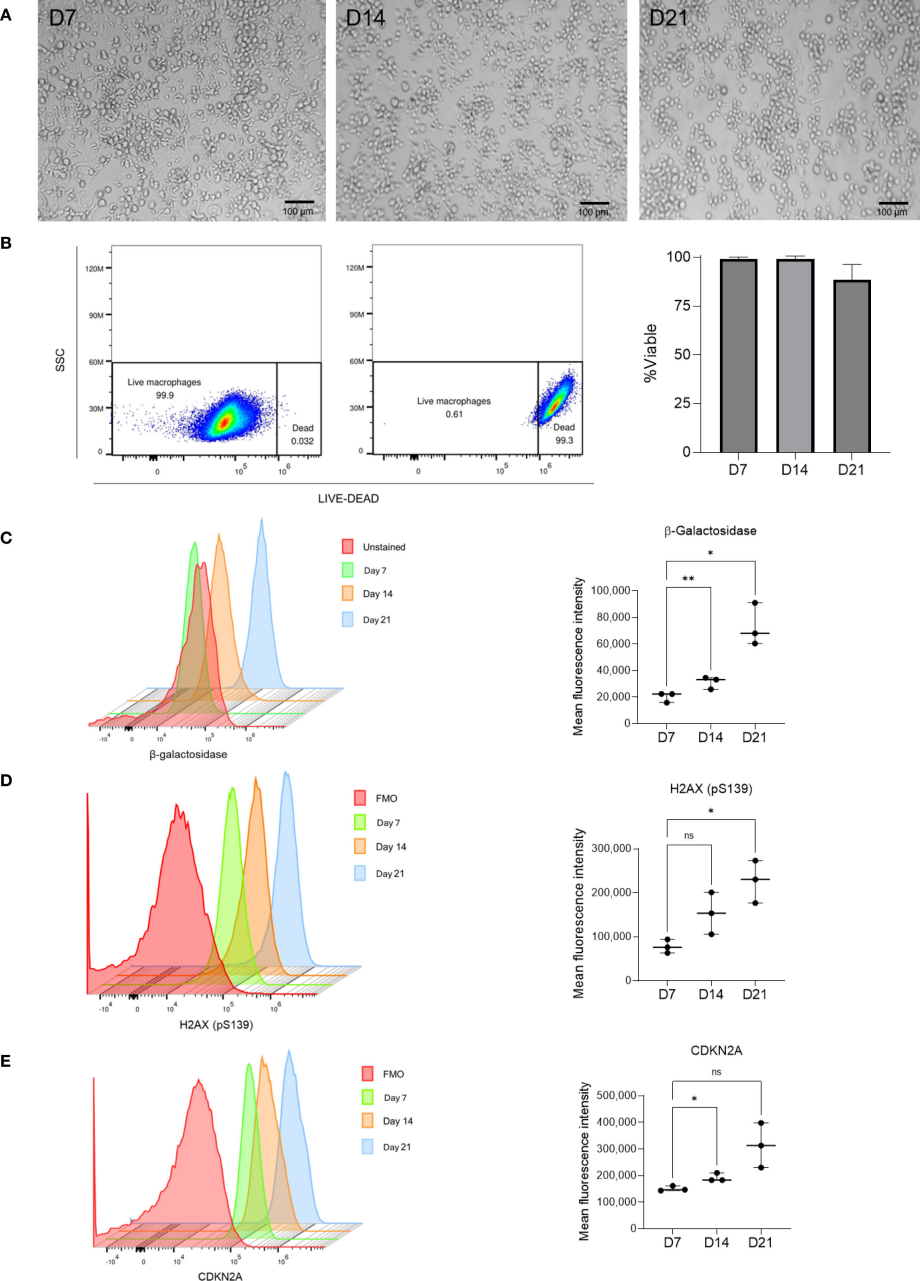
Induction of macrophage senescence through long-term *in vitro* culture. **(A)** Brightfield images show morphological changes in monocyte-derived macrophages at days 7, 14, and 21, consistent with senescence progression (scale bar = 100 μm). **(B)** Viability was assessed using LIVE/DEAD staining. Cell viability remained >95% at all time points, with a slight decline at day 21; the depicted values represent the mean ± SEM from n = 3 independent donors. (c–e) Mean fluorescence intensity (MFI) of β-galactosidase **(C)**, H2AX (pS139) **(D)**, and CDKN2A **(E)** increased significantly over time. Normality was assessed with the Shapiro–Wilk test. Statistical significance was determined using repeated-measures one-way ANOVA with Dunnett’s multiple comparisons test. ns: not significant (p ≥ 0.05), *p < 0.05, **p < 0.01. Individual data (n=3) with median and interquartile ranges are depicted.

We established an *in vitro* long-term culture model to investigate cellular senescence in human macrophages. Peripheral blood monocytes were isolated and differentiated into monocyte-derived macrophages (MDMs) for seven days (D7), 14 (D14), and 21 (D21) days (**Figure 1A**). Brightfield microscopy revealed progressive morphological changes, including increased cell size, flattening, and granularity, hallmarks commonly associated with senescent cells.

Cell viability was evaluated across all time points and analyzed by flow cytometry using heat-killed macrophages as a reference. We observed that most macrophages remained viable, with a death rate consistently below 5% at D7, D14, and D21. This indicated that the extended culture conditions were not cytotoxic and supports the model’s suitability for long-term studies (**Figure 1B**).

Flow cytometric analysis showed a marked, time-dependent increase in the mean fluorescence intensity (MFI) of senescence-associated β-galactosidase (SA-β-gal), the cyclin-dependent kinase inhibitor CDKN2A (p16INK4a), and phosphorylated histone H2AX (H2AXpS139), a marker of the DNA damage response activation between D7, D14, and D21 (**Figures 1C–E**). Statistical comparisons confirmed significant upregulation of CDKN2A at both D14 (p = 0.012) and D21 (p = 0.001), along with significant increases in β-galactosidase and H2AX levels (p < 0.05).

### 3.2 Phenotypic shifts in senescent macrophages identified by high-dimensional clustering

The phenotypical changes and activation state in macrophage was assessed by multiparametric flow cytometry focus on features of monocyte-to-macrophage differentiation (CD14 and CD64), anti-inflammatory M2-like phenotypes (CD206 and CD163), antigen presentation (HLA-DR), innate immune sensing (TLR2 (CD282) and TLR9 (CD289), and senescence-associated molecular responses (H2AX, and CDKN2A).

**Figure 2:**
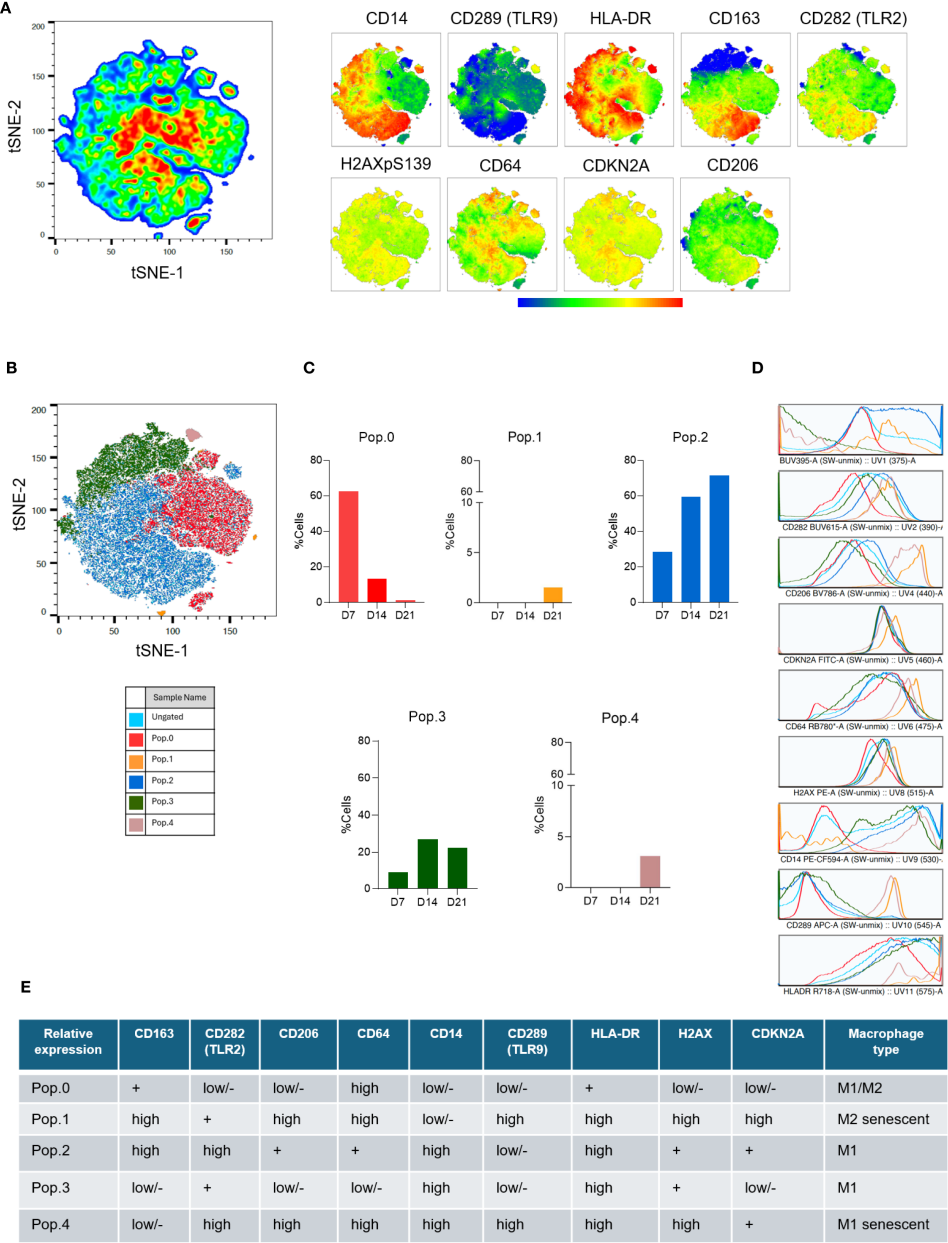
Phenotypic remodeling of macrophages during senescence. **(A)** t-SNE projections display marker expression profiles (CD14, CD289 (TLR9), HLA-DR, CD163, CD282 (TLR2), H2AXpS139, CD64, CDKN2A and CD206) at D7, D14, and D21 (n = 3 donors; 521,000 concatenated events). **(B)** FlowSOM analysis identified five metaclusters (Pop.0–Pop.4) with distinct temporal dynamics. **(C)** Cluster frequency analysis revealed a reduction in Pop.0 and an increase in Pop.2 and Pop.3 by D21. Depicted is the frequency of all the concatenated events. **(D)** Histograms show marker expression per cluster; relative marker intensity was used to define pro- or anti-inflammatory phenotypes. **(E)** The Summary table classifies each cluster based on marker expression as M1, M2, or senescent-like macrophages. The analysis is based on live, unstimulated cells from three donors.

Using t-SNE for dimensionality reduction, we concatenated 521,000 live-single cell events on D7, D14, and D21 (n = 3 donors). The t-SNE maps revealed considerable heterogeneity in marker expression and notable temporal remodeling of the macrophage landscape (**Figure 2A**). Unsupervised FlowSOM clustering stratified the population into five metaclusters (Pop.0 to Pop.4), each displaying distinct immunophenotypes (**Figure 2B**).

Quantitative analysis of cluster abundance over time showed a marked reduction in Pop.0, from 60% at D7 to approximately 3% at D21, accompanied by a progressive expansion of Pop.2 and Pop.3, which together represented over 90% of cells by D21 (**Figure 2C**). Histograms and tabulated marker summaries revealed that early clusters (Pop.1; Pop.2) expressed high levels of CD206 and CD163, consistent with an M2-like anti-inflammatory phenotype, whereas late-stage clusters (Pop.3; Pop.4) exhibited elevated CD14, CD64, and TLR2 levels, hallmarks of a proinflammatory M1-like state (**Figures 2D, E**). Pop.4 also exhibited high expression of HLA-DR and TLR9. Thus, in addition to the expression of molecular markers of senescence, our aged macrophages undergo functional reprogramming toward a proinflammatory, less plastic state.

### 3.3 Senescent macrophages exhibit an M1-like phenotype upon LPS stimulation

To evaluate how macrophages at different stages of senescence respond to inflammatory stimuli such as LPS. We applied the T-REX algorithm to t-SNE projections to assess the resulting changes in cellular phenotypes.

**Figure 3:**
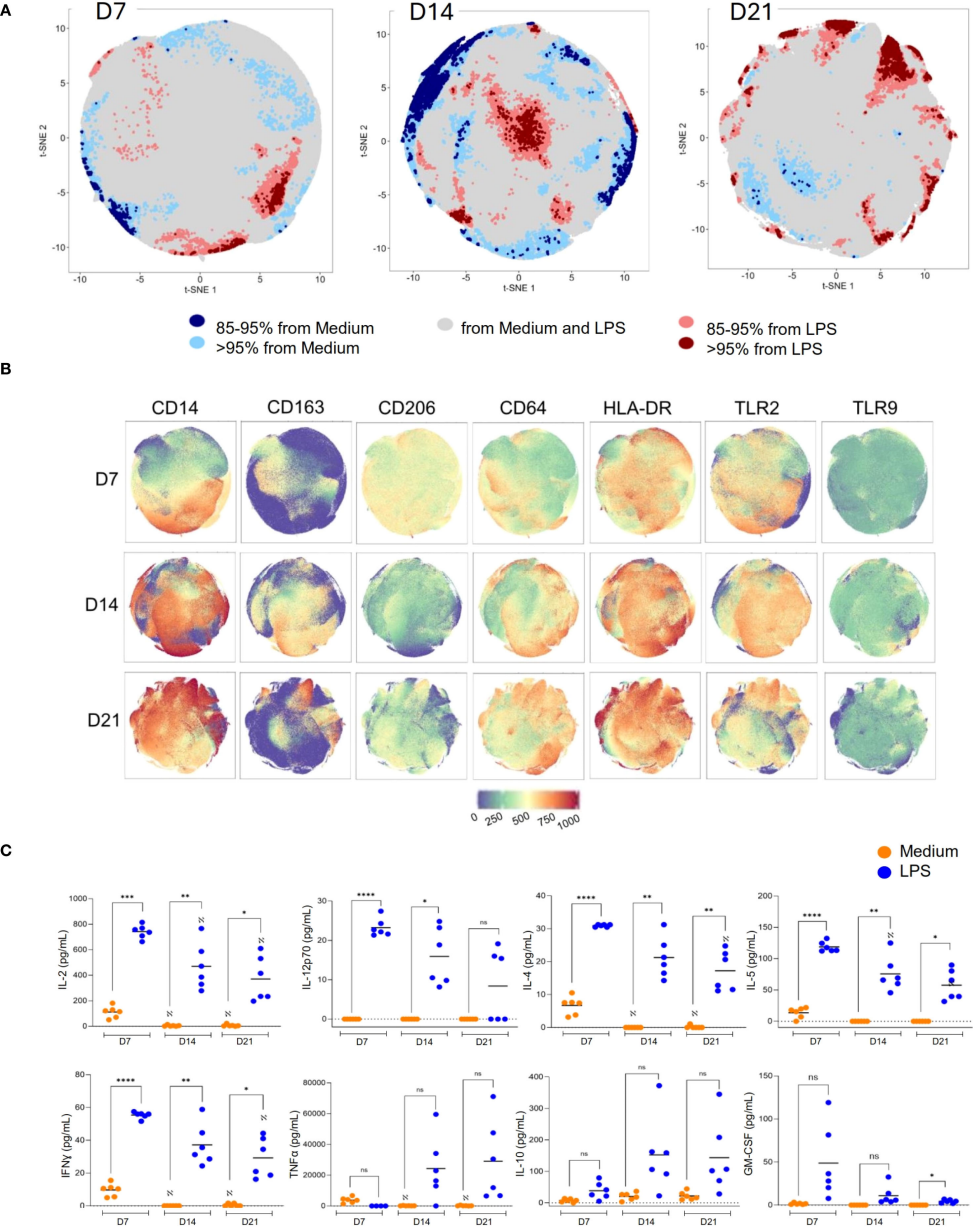
Functional dysregulation in senescent macrophages upon LPS stimulation. **(A)** T-REX analysis of t-SNE plots shows distinct clustering of LPS-stimulated vs. unstimulated macrophages at D7, D14, and D21. At D21, senescent macrophages formed discrete LPS-enriched clusters (>95% of events per region). **(B)** CD14, CD64, and TLR2 expression was increased in senescent macrophages (D21) upon LPS stimulation, while CD206, CD163, and TLR9 were reduced, consistent with M1-like polarization (n = 3 donors). **(C)** Cytokine secretion was measured after 6 h of LPS stimulation, including IL-2, IL-12p70, IL-4, IL-5, IFN-γ, TNF-α, IL-10, and GM-CSF compared to medium. Normality was assessed with the Shapiro–Wilk test. For paired comparisons, the Wilcoxon matched-pairs signed rank test was applied. *p < 0.05, **p < 0.01, ***p < 0.001, ****p < 0.0001. Data represent individual results with means from n = 6 independent donors.

At day 7, t-SNE projections revealed that most macrophages were distributed within gray regions, indicating a high degree of phenotypic overlap between unstimulated and LPS-treated conditions and suggesting a homogeneous activation response (**Figure 3A**). In contrast, distinct red clusters emerged by D14, and more prominently at D21, representing phenotypic regions where >95% of events originated from LPS-stimulated cells. These red clusters indicate the presence of LPS-responsive subpopulations unique to senescent macrophages.

Phenotypic characterization of these LPS-enriched clusters showed increased CD14, CD64, and TLR2 expression and a consistent downregulation of CD206, CD163, and TLR9 (**Figure 3B**).

### 3.4 Senescent macrophages display a dysregulated and impaired cytokine profile

To investigate the cytokine secretion from macrophages cultured for 7, 14, and 21 days, we analyzed the supernatants of LPS-stimulated macrophages collected after 24 hours of stimulation. On day 7 (D7), macrophages responded robustly to LPS stimulation, producing high levels of IL-12p70, IL-4, IL-5, IL-2, and IFN-γ, consistent with an immunocompetent, plastic functional profile (**Figure 3C**).

**Figure 4:**
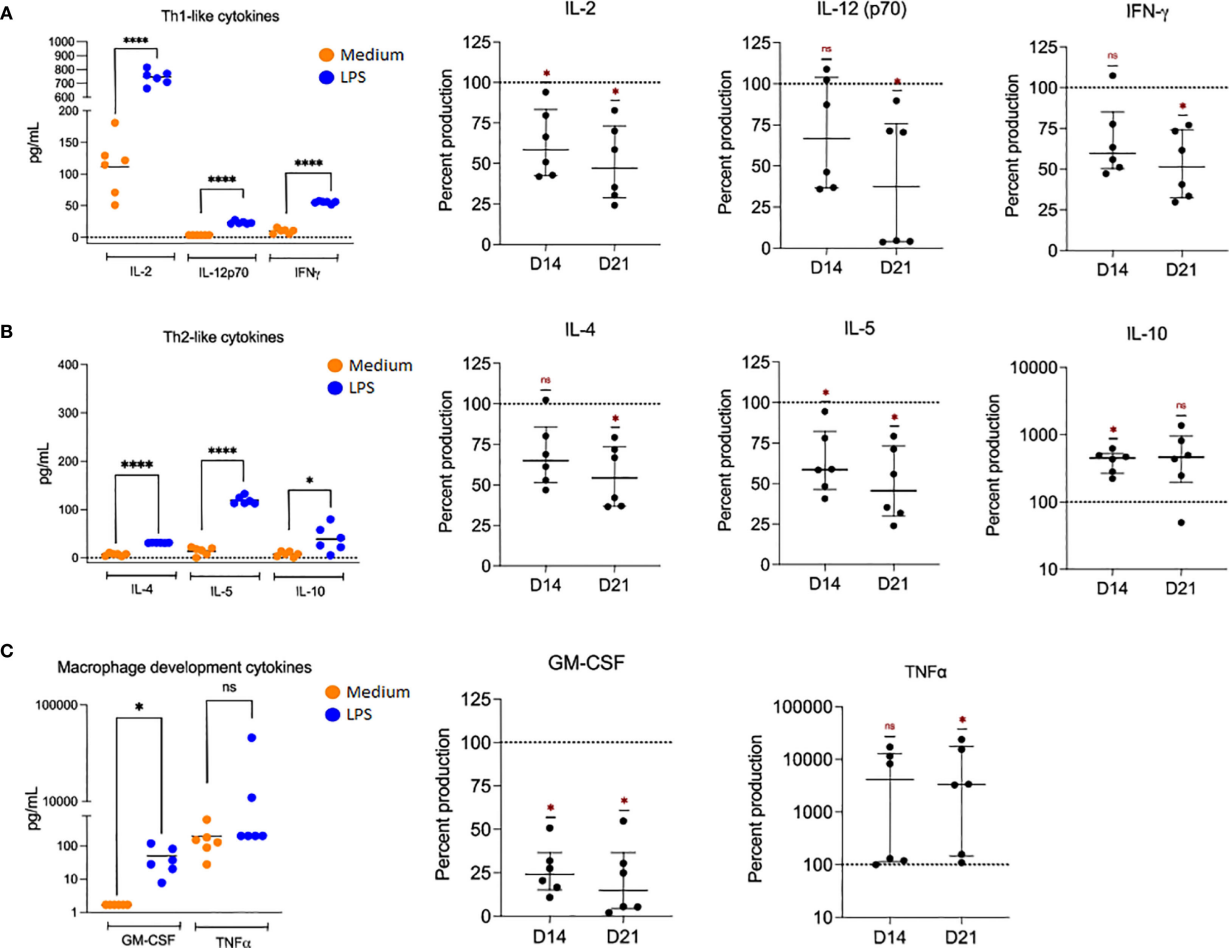
Cytokine secretion profiles of senescent macrophages upon LPS stimulation. Macrophages were cultured for 6 h in the presence of medium or LPS. **(A)** Secretion of Th1-type cytokines IL-2, IL-12p70, and IFN-γ in macrophages cultured for 7 days (D7, left) and stimulated with either medium or LPS. Cytokine percent production, relative to D7, was quantified at D14 and D21 (right). **(B)** Th2-like cytokine production of IL-4, IL-5, and IL-10 was measured at D7 (left), and the relative percent production of these cytokines was quantified at D14 and D21 (right). **(C)** Macrophage development cytokines, GM-CSF and TNF-α, were measured at D7 (left), and the relative percent production of these cytokines was quantified at D14 and D21 (right). Normality was tested with the Shapiro–Wilk test, followed by repeated-measures one-way ANOVA with Dunnett’s multiple comparisons test. *p < 0.05, ****p < 0.0001. Data represent individual results with means (left) or medians with interquartile range (right) from n = 6 independent donors.

In contrast, macrophages cultured for 21 days (D21) exhibited a markedly restricted cytokine output. These senescent cells secrete significantly reduced amounts of IL-2, IFN-γ, IL-4, IL-5, IL-12p70, but increased amounts of IL-10 and TNF-α (**Figures 3C**, **4**), suggesting a dysregulated immune profile consistent with the senescence-associated secretory phenotype (SASP), as previously described (10, 12). These findings underscore how macrophage senescence compromises phenotypic identity and effector function, potentially contributing to age-associated immune dysfunction.

## 4 Discussion

This study presents a robust and scalable *in vitro* model that successfully portrays macrophage senescence, allowing for a comprehensive analysis of phenotypic and functional dysregulation associated with aging. By extending monocyte-derived macrophage (MDM) cultures up to 21 days, we observed hallmark features of cellular senescence, including increased expression of SA-β-gal, CDKN2A (p16INK4a), and H2AXpS139, with sustained cell viability. These results are consistent with canonical markers of irreversible growth arrest, lysosomal activation, and DNA damage response pathways, thereby validating the model’s biological relevance (3, 10, 16, 21). Additional metrics, such as 000000CDKN1A expression, mitochondrial membrane potential, reactive oxygen species (ROS), and metabolic profiling, could enhance the resolution of distinct senescence trajectories (25–27).

Our high-dimensional flow cytometry data revealed dynamic remodeling of macrophage identity over culture time. Macrophages comprised mainly two populations, one with a plastic M1/M2 phenotype (Pop.0), and one with an M1 phenotype (Pop.2) at Day 7 (D7). In contrast, macrophages shifted toward a CD14+/CD64+/TLR2+ phenotype associated with M1-like activation and microbial sensing with the introduction of senescent M1 and M2 macrophages (Pop.1 and Pop.4) on 21 days of culture (D21). This phenotypic transition suggests that macrophage senescence reflects cellular aging and skews polarization toward an inflammatory and less plastic state. Such M1-like skewing is aligned with previous observations of innate immune reprogramming in aging and may underpin chronic inflammation in older individuals (22–24). This restricted activation pattern may contribute to chronic, unresolved inflammation in aging tissues.

Functionally, senescent macrophages exhibit a restricted cytokine response upon LPS stimulation, significantly downregulating IL-2, IFN-γ, IL-12p70, IL-4, IL-5, and increasing production of TNF-α and GM-CSF. This imbalance reflects a partially active but dysfunctional phenotype, characteristic of the senescence-associated secretory phenotype (SASP) (11, 12, 17). Notably, the increase in TNF-α may have a role in senescent macrophage-derived inflammation; meanwhile, the increased IL-10 secretion suggests a compensatory, possibly maladaptive anti-inflammatory feedback loop that may suppress protective immune responses or facilitate immune evasion. Together with the phenotype results, these data demonstrate that senescent macrophages retain the capacity to respond to bacterial stimuli but do so in a limited and skewed manner, favoring a dysregulated M1-like phenotype and reduced functional plasticity.

One of the advantages of our model is the lack of additional chemical or physical stressors to induce senescence, which may obscure the interpretation of the macrophages’ phenotype and innate responses. Also, the use of flow cytometry for the detection of senescence cytosolic and nuclear factors at the single-cell level enabled the simultaneous analysis with surface and intracellular markers while maintaining compatibility with high-throughput platforms. Moreover, the spectral cytometer also automatically subtracted the autofluorescence of the macrophages, which is a challenge for conventional cytometric evaluations. Such cytometry-compatible assays are essential for dissecting heterogeneity in senescent cell populations and correlating function with phenotype.

Although our study provides phenotypic and cytokine-based functional analyses, it is important to acknowledge certain limitations and avenues for future research. For instance, we did not directly assess the phagocytic capacity of macrophages or their production of reactive oxygen species (ROS). The phagocytic ability of macrophages, the process by which they engulf pathogens, cellular debris, and foreign particles, is a fundamental component of the innate immune response, and its decline is a hallmark of immune aging. Similarly, ROS production is essential for eliminating pathogens and modulating inflammatory responses. Dysregulated ROS production, however, can contribute to oxidative stress and tissue damage, which are key drivers of chronic inflammation. Therefore, future studies could incorporate these assessments to evaluate the quality of the immune response. Also, to gain a deeper mechanistic understanding, future studies could integrate transcriptomic and proteomic profiling, as well as metabolic characterization, to identify key regulators and pathways involved in macrophage senescence (28–31).

Our results may explain the link between macrophage senescence and the immunological hallmarks of aging, particularly inflammaging, a chronic, sterile, low-grade inflammatory state implicated in multiple age-related pathologies, including atherosclerosis, metabolic syndrome, and neurodegeneration. Although this *in vitro* model captures several hallmark features of macrophage senescence, certain limitations should be acknowledged. Donor-to-donor variability can introduce biological heterogeneity that may obscure subtle phenotypic shifts. Furthermore, the absence of *in vivo* environmental cues, such as systemic factors, extracellular matrix components, and paracrine signaling, limits the model’s physiological relevance. Also, in future research, a direct comparison with PBMCs from elderly donors would provide additional validation. However, this controlled and reproducible system makes it well-suited for mechanistic exploration and intervention testing. Importantly, it allows for the standardized evaluation of senescence-targeting strategies, such as therapeutics, immunomodulators, or metabolic reprogramming agents (32, 33). Moreover, it could serve as a preclinical platform for assessing vaccine efficacy or adjuvant responses in aged immune contexts, contributing to personalized and geriatric immunology.

In summary, our findings demonstrate that extended *in vitro* culture of human macrophages induces a senescent state that mirrors key aspects of *in vivo* immunosenescence. The model integrates temporal, phenotypic, and functional dimensions, offering a valuable tool to explore the role of senescent macrophages in age-related inflammation and immune decline. By facilitating the identification of molecular targets and therapeutic strategies, this model contributes to the growing field of immunosenescence and supports efforts to mitigate inflammaging and restore immune resilience in aging populations.

## 5 Conclusion

This study establishes a robust and physiologically relevant model to investigate the onset and consequences of macrophage senescence. By extending monocyte-derived macrophage (MDM) cultures beyond the conventional 7-day differentiation period to 14 and 21 days, we successfully induced a senescent phenotype. We identified a progressive remodeling of surface marker expression profiles using high-dimensional flow cytometry combined with unsupervised clustering algorithms. This remodeling involved a shift toward proinflammatory states, reflecting a loss of immunological plasticity associated with cellular aging. This model provides a versatile tool for the in-depth study of macrophage senescence, integrating phenotypic, temporal, and functional aspects. It may facilitate the identification of novel biomarkers of immune aging, support the development of senescence-targeted therapies, and contribute to future strategies aimed at counteracting inflammaging and restoring immune competence in aged populations.

## Data Availability

The original contributions presented in the study are included in the article/**Supplementary Material**. Further inquiries can be directed to the corresponding author.
