# Supplementary material for: A long-term culture model for investigating senescence-associated dysregulation in macrophages
Source: Front Immunol. 2025 Oct 2;16:1661497. doi: 10.3389/fimmu.2025.1661497 (PMC12529554; doi:10.3389/fimmu.2025.1661497)
Supplement: Supplementary file 1 [file Supplementaryfile1.docx]

A Long-Term Culture Model for Investigating Senescence-Associated Dysregulation in Macrophages

Andy Ruiz, María Guadalupe Lucero-Gil, Martha Torres, Esmeralda Juárez

^
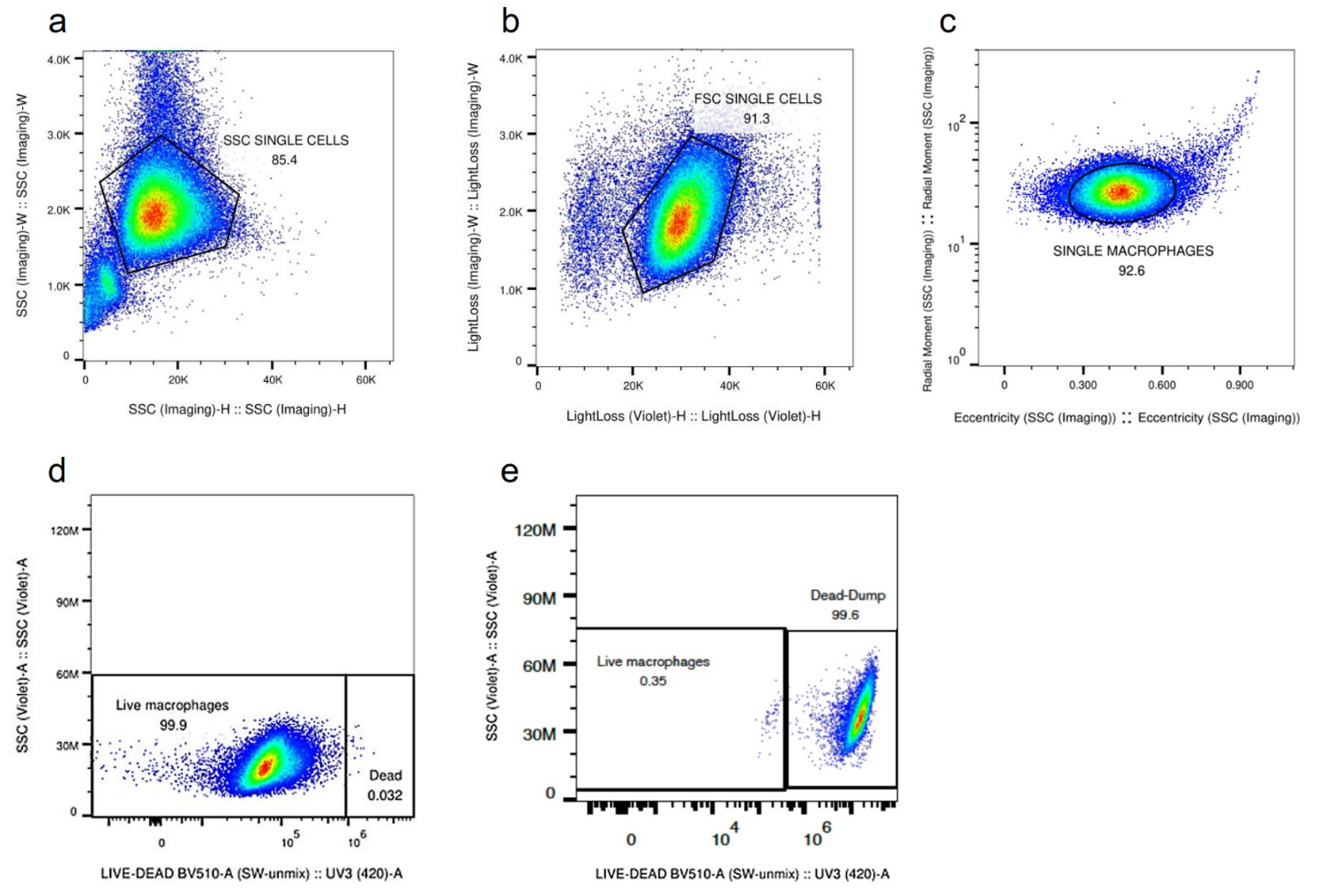
^

**Supplementary Figure 1.** **Flow cytometry gating strategy for identification of viable single macrophages.** (a–b) Selection of single cells based on SSC-H vs. SSC-W and LightLoss-H vs. LightLoss-W parameters. (c) Gating of single CD14+ macrophages based on their eccentricity vs radial moment. (d) Viability gating using LIVE/DEAD BV510; viable cells represent >95% of the total. The Dead-Dump gate included death cells and those positive for CD3, which was measured with the same detector. (e) Heat-killed macrophages were used to confirm the accuracy of gating. This strategy ensured consistent selection of viable and phenotypically representative macrophage populations across all time points.

**Supplementary Table 1. Antibodies and Reagents Used for Flow Cytometry Analysis**

| **Antibody/Reagent** | **Fluorochrome/Cat. Number** | **Clone** | **Dilution** | **Brand** | **RRDI** |
| --- | --- | --- | --- | --- | --- |
| CD3 | BV510 / 568556 | UCHT1 | 1:34 | BD Horizon | AB_2941961 |
| CD14 | PE-CF594 / 562335 | MΦP9 | 1:34 | BD Horizon | AB_11153663 |
| CD64 | RB780 / 569213 | 10.1 | 1:34 | BD Horizon | AB_2869007 |
| CD163 | BUV395 / 568191 | MAC2-158 | 1:34 | BD Horizon | AB_2743092 |
| CD206 | BV786 / 751773 | 15-2 | 1:34 | BD OptiBuild | AB_2740622 |
| HLADR | R718 / 568579 | L243 | 1:34 | BD Horizon | AB_3684385 |
| CD282 (TLR2) | BUV615 / 751484 | 11G7 | 1:34 | BD OptiBuild | N/A |
| CD289 (TLR 9) | APC / 560428 | eB72-1665 | 1:27 | BD Pharmingen | AB_1645622 |
| CDKN2A/p16-INK4a | FITC / BS-23797R-FITC | Polyclonal | 1:30 | Thermo Fisher Scientific | N/A |
| H2AX | Pe / 562377 | N1-431 | 1:30 | BD Pharmingen | AB_2737611 |
| 7AAD | 559925 | - | 1:4 | BD Pharmingen | AB_2869266 |
| CD14 MicroBeads | 130-050-201 | - | - | Miltenyi Biotec (USA) | AB_2665456 |
| CellEvent Senescence Green Flow Cytometry Assay Kit | C10841 | - | 1:100 | Thermo Fisher Scientific | AB_2737611 |
| LIVE/DEAD Fixable Dead Cell Stain Kits | L34957 | - | 1:1000 | Thermo Fisher Scientific | N/A |
| BD Cytofix/Cytoperm | 554714 | - | - | BD | AB_2869008 |
| Brilliant Stain Buffer | 566349 | - | - | BD Horizon | AB_2869750 |

N/A: not registered at time of publication.

**Supplementary Table 2. Reagents, Stimuli, Kits, and Software**

| **Reagent** | **Cat. Number** | **Working Concentration** | **Brand (Country)** | **Identifier (RRDI)** |
| --- | --- | --- | --- | --- |
| Lymphoprep™ (PBMC isolation) | 1114547 | - | Axis Shield (Germany) | N/A |
| RPMI 1640 medium | 12-167F | - | Lonza (USA) | N/A |
| L-Glutamine 200 mM | 17-605E | 200 mM | Lonza (USA) | N/A |
| Fetal Bovine Serum (FBS), heat-inactivated | 14-501F | 10% | Lonza (USA) | N/A |
| Penicillin-Streptomycin (100X) | 17-602E | 1% | Lonza (USA) | N/A |
| Paraformaldehyde 2% | 158127 | 2% | Sigma-Aldrich (USA) | N/A |
| Bovine Serum Albumin (BSA) | A7906 | 1% | Sigma-Aldrich (USA) | N/A |
| LPS (E. coli O111:B4) | L4391 | 200 ng/mL | Sigma-Aldrich (USA) | N/A |
| Bio-Plex Pro™ Human Cytokine Panel | M50000007A (8-plex, IL-2, IL-4, IL-5, IL-10, IL-12p70, IFN-γ, TNF-α, GM-CSF) | - | Bio-Rad (USA) | N/A |
| Bio-Plex Manager™ software | - | - | Bio-Rad | N/A |
| FlowJo™ v10.10 software | - | - | BD Biosciences | SCR_008520 |
| GraphPad Prism Software v10 | - | - | GraphPad Software | SCR_002798 |

N/A: not applicable or not registered at time of publication.

**Supplementary Table 3. Cytokine Detection Limits According to Manufacturer’s Specifications**

| **Cytokine** | **Quantification range (pg/mL)** | **Limit of Detection (LOD, pg/mL)** |
| --- | --- | --- |
| IL-2 | 1 – 2,000 | 1.0 |
| IL-4 | 0.5 – 1,000 | 0.5 |
| IL-5 | 1 – 2,000 | 2.0 |
| IL-10 | 1 – 2,000 | 2.0 |
| IL-12p70 | 1 – 2,000 | 2.5 |
| IFN-γ | 1 – 5,000 | 2.0 |
| TNF-α | 1 – 5,000 | 6.0 |
| GM-CSF | 1 – 2,000 | 2.0 |
